# Stereometric parameters change vs. Topographic Change Analysis (TCA) agreement in Heidelberg Retina Tomography III (HRT-3) early detection of clinical significant glaucoma progression

**Published:** 2014

**Authors:** AM Dascalu, AP Cherecheanu, D Stana, L Voinea, R Ciuluvica, C Savlovschi, D Serban

**Affiliations:** * ”Carol Davila” University of Medicine and Pharmacy, Bucharest, Romania; ** University Emergency Hospital, Bucharest, Romania

**Keywords:** stereometric parameters, Heidelberg retina tomography, glaucoma progression

## Abstract

**Purpose:** to investigate the *sensitivity* and *specificity* of the stereometric parameters change analysis vs. Topographic Change Analysis in early detection of glaucoma progression.

**Methods:** 81 patients with POAG were monitored for 4 years (GAT monthly, SAP at every 6 months, optic disc photographs and HRT3 yearly). The *exclusion criteria were* other optic disc or retinal pathology; topographic standard deviation (TSD >30; inter-test variation of reference height > 25 μm. The *criterion for structural progression was the following:* at least 20 adjacent super-pixels with a clinically significant decrease in height (>5%).

**Results:** 16 patients of the total 81 presented structural progression on TCA. The *most useful stereometric parameters* for the early detection of glaucoma progression were the following: Rim Area change (sensitivity 100%, specificity 74.2% for a “cut-off ” value of -0.05), C/D Area change (sensitivity 85.7%, specificity 71.5% for a “cut off ” value of 0.02), C/D linear change (sensitivity 85.7%, specificity 71.5% for a “cut-off ” value of 0.02), Rim Volume change (sensitivity 71.4%, specificity 88.8% for a “cut-off ” value of -0.04). *RNFL Thickness* change (<0) was highly sensitive (82%), but less specific for glaucoma progression (45,2%). Changes of the other stereometric parameters have a limited diagnostic value for the early detection of glaucoma progression.

**Conclusion:** TCA is a valuable tool for the assessment of the structural progression in glaucoma patients and its inter-test variability is low. On long-term, the quantitative analysis according to stereometric parameters change is also very important. The most relevant parameters to detect progression are RA, C/D Area, Linear C/D and RV.

## Introduction

Despite the continuous development of the imagistic techniques and statistically based software of analysis, the early detection of the clinically significant structural progression is still a challenge today in the management of the glaucomatous patients. Heidelberg Retina Tomograph III (HRT-3) identifies the structural changes of the optic disk and peripapillary retina according to 2 event-based algorithms: stereometric parameters change analysis and Topographic Change Analysis (TCA). The normalized changes graphics are generated based on the variation of the stereometric parameters measured for at least 2 consecutive exams for the same subject, and estimate the trend of the losses of the optic nervous fibers along time. The clinical relevance of these 3 types of analysis generated by HRT-3 software is not yet fully investigated and there is no gold-standard or a minimal set of criteria considered to define the clinical significance of a structural change identified by the machine.

Furthermore, the data of the 3 algorithms for the detection of progression are not always concordant. An explanation of this fact is that these are in different amounts affected by the factors that induce inter-test variability in the evaluation of the optic disc and peripapillary retina. Topographic Change Analysis (TCA) is considered the least influenced by interest variability, as it is independent of the definition of the optic disc contour, and, though, it is not affected by the setting of the reference plan (and the variation of this parameter between 2 examinations). The reference plan, a parameter that is automatically calculated by the machine in every optic disc scan achieved. Test-retest variation of the reference plan has an important effect on the stereometric change analysis, as all the stereometric parameters are calculated in relation to it.

The present study investigates the *sensitivity* and *specificity* of the stereometric parameters change analyses vs. Topographic Change Analyses (TCA) in the early detection of glaucoma progression.

## Materials and method

The study evaluated a group of 81 patients with POAG, who were monitored for 4 years, by Goldmann applanation tonometry (GAT) monthly, standard automated perimetry (Optopol PTS-910, Glaucoma program, Fast threshold strategy) at every 6 months, optic disc photographs (VisuCam Zeiss) and Heidelberg Retina tomography (HRT-3) yearly.

The ***inclusion criteria*** were the following:

- age >35 years;

- diagnosis of POAG according to the European Glaucoma Society Guidelines (3rd edition);

The ***exclusion criteria*** were the following:

- optic disc or retinal pathology that might interfere with the detection of glaucoma progression;

- topographic standard deviation (TSD) >30 , as a quality index for HRT exams;

- inter-test variation of reference height (RH) above 25 μm for HRT exams taken for the same subject;

As several previous studies have proved the direct impact of the interest variability of the reference height upon the stereometric parameters change, we chose the criteria as a quality index for relevance of stereometric change analysis [**2**,**3**,**5**].

The criterion for structural progression was considered to be of at least 20 adjacent super-pixels with clinically significant decrease in height (>5%).

The sensitivity and sensibility of the stereometric parameters change analysis were evaluated while considering the above-mentioned criteria for TCA as golden-standard for the structural progression. The difference between the initial and the final value for each parameter was analyzed in correlation with the diagnosis of progression made by TCA. The diagnosis quality of each parameter in terms of sensitivity and specify was evaluated by using the ROC curves (receiver operating characteristic curve), as the relation between sensibility (represented on X-axis) and 1- specificity (represented on Y axis) of the test.

## Results

16 patients of the total 81 presented structural progression on TCA, according to the criteria for progression used in this study and 67 were considered to present no change.

The most useful stereometric parameters for the early detection of glaucoma progression were the following: Rim Area change, C/D Area change, C/D linear change, Rim Volume change. The sensibility and specificity at the “cut-off” value is presented in the following table:

**Table 1:** Comparative sensitivity and specificity at the “cut-off” value for the most important stereometric parameters

	“Cut-off” value	Sensitivity	Specificity
RA	<-0,05	100%	74,2%
RV	<-0,04	71,4%	88,8%
C/D area	>0,02	85,7%	71,5%
C/D linear	>0,02	85,7%	78,1%
RNFLT	<0	100%	45,7%

RNFL Thickness change (<0) was highly sensitive (82%), but less specific for glaucoma progression (45,2%).

Changes of the other stereometric parameters (Height variation contour - HVC, cup shape measure - CSM, mean cup depth and maximum cup depth) have a limited diagnostic value for the early detection of glaucoma progression.

**Graph 1:**
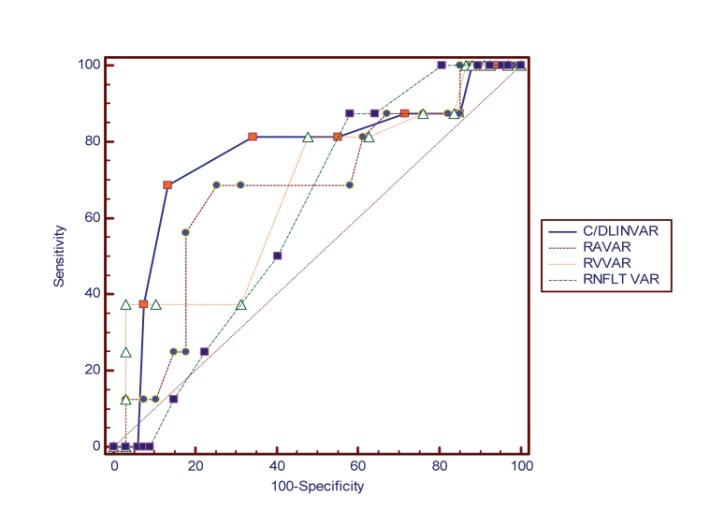
Comparative ROC (sensitivity vs 1-specificity) for C/D linear change, RA change, RV change, RNFLT change

**Table 2:** Comparative AUC (area under curve) for the investigated stereometric parameters change

	**AUC**	**SE a**	**95% CI b**
**C/ DLINVAR**	0,764	0,0765	0,659 to 0,851
**RAVAR**	0,676	0,0781	0,565 to 0,775
**RVVAR**	0,666	0,0787	0,554 to 0,766
**RNFLT_VAR**	0,608	0,0645	0,495 to 0,71
**CSMVAR**	0,577	0,0769	0,464 to 0,685
**HVCVAR**	0,500	0,0845	0,389 to 0,612
**C/D AREAVAR**	0,751	0,0764	0,644 to 0,840
a DeLong et al., 1988			
b Binomial exact			

## Discussions and conclusions

Several studies have compared the performances of Heidelberg Retina Tomography in the early detection of glaucomatous progression, in relation to different other structural or functional tests [**1**-**4**,**6**,**7**]. There is though very little information in the reviewed literature regarding the clinical value of the stereometric parameters change analysis. The present study used the Topographic Change Analysis as a gold-standard for the detection of progression, with at least 20 adjacent super pixels with statistically significant (<5%) decrease in height, the most widely used in clinical practice when assessing progression [**2**,**5**,**8**].

Among the HRT software statistic analysis tools, **Topographic Change Analysis** is the most useful in immediate management of the glaucomatous patient, as it is the least affected by the inherent test-retest variability generated by the machine (TSD and reference height variability). Due to the graphic representation of the location and area with changes in heights, TCA is very easy to use in clinical practice [**1**-**3**,**8**].

On long-term, quantitative analysis according to stereometric parameters change is also very important.

In the present study, the most relevant parameters to detect progression proved to be rim area (RA), area cup/disc ratio (C/D Area), Linear cup/disc ratio (linear C/D) and rim volume (RV).

As all the further examinations are compared to baseline, the detection of progression is influenced by the quality of data registered during the first exam. Setting a correct optic disc contour, a good quality of image (low TSD<30) and taking 2-3 scans during the same examination to estimate the interest variability for the examined patient are very important for a good long term follow-up [**3**,**5**,**9**]. Searching for a good lineation of the compared images and a low interest variation of the reference height are 2 other important aspects to have in mind in order to increase the clinical significance of the automated generated results.

Due to the generally slowly progression of the IOP compensated glaucoma and the relatively-recent introduction of the statistical based algorithms of detecting progression in ophthalmological practice, further long-term prospective studies are necessary to define the clinical relevance of the results.
